# FK506 binding protein 12.6-mediated inhibition of sperm-specific calcineurin is essential for FK506-induced male infertility by disturbing the homeostasis of calcium and mitochondria

**DOI:** 10.1186/s43556-025-00391-3

**Published:** 2025-12-22

**Authors:** Yun-Fei Xiao, Shi-Fen Yang, Shi-Ang Huang, Zhi-Xiong Zeng, Li-Na Gong, Lin Xie, Ling-Fang Wang, Xiao-Hui Guan, Mei-Xiu Jiang, Yi-Song Qian, Ke-Yu Deng, Hong-Bo Xin

**Affiliations:** 1 National Engineering Research Center for Bioengineering Drugs and the Technologies, Institute of Translational Medicine, Jiangxi Medical College, Nanchang University, Nanchang, People’s Republic of China; 2 Jiangxi Province Key Laboratory of Bioengineering Drugs, Institute of Translational Medicine, Nanchang, People’s Republic of China; 3 School of Pharmacy, Jiangxi Medical College, Nanchang University, Nanchang, 330031 People’s Republic of China; 4 Jiangxi Provincial People’s Hospital, the First Affiliated Hospital of Nanchang Medical College, Nanchang, 330031 People’s Republic of China

**Keywords:** Male infertility, FKBP12.6, Calcineurin, Calcium release, Mitochondria

## Abstract

Impaired sperm motility is a leading cause of male infertility. Studies indicated that FK506, an immunosuppressive drug, resulted in male mouse infertility or an overall decline in the fertilization capacity of male renal transplant recipients. However, the underlying mechanism is not fully elucidated. Here, we reported that the disruption of FK506 binding protein 12.6 (FKBP12.6) significantly alleviated FK506-induced male infertility in mice by restoring sperm motility and mitochondrial functions in immature sperm. Mechanically, we identified that the FK506-FKBP12.6 complex preferentially bound to the sperm-specific calcineurin, which is composed of a catalytic subunit (PPP3CC) and a regulatory subunit (PPP3R2). We revealed that FKBP12.6 deficiency reversed FK506-induced the elevated expression of Down syndrome critical region 1.1 (DSCR1.1, a calcineurin inhibitor) and the reduced expressions of PPP3CC and PPP3R2 in immature sperm. Additionally, we observed that FKBP12.6 deficiency remarkably improved FK506-induced the abnormality of Ca^2+^ release through restoring calcineurin-mediated dephosphorylation at S2808 and S2814 of RyR2, and maintained the mitochondrial homeostasis by suppressing the hyperphosphorylation at S637 of the mitochondrial dynamin-related protein 1 (Drp1) in immature sperm. Furthermore, we demonstrated that FKBP12.6 deficiency reversed FK506-induced the reduction of the acrosome reaction in sperm by retaining the expression of synaptosome-associated protein of 25 kDa (Snap25) in immature sperm, which is essential for the maturation of acrosome exocytosis function. Certainly, our findings should provide an insight in elucidating the mechanism of FK506-induced male infertility, suggesting that FKBP12.6 might be a potential target for male infertility clinically.

**Supplementary Information:**

The online version contains supplementary material available at 10.1186/s43556-025-00391-3.

## Introduction

Infertility is a major health problem that affects 8–12% of couples globally [1, 2], among which male infertility accounts for 50% of the total infertility [3, 4]. Sperm is generated in testes and matured in epididymal ducts including the proximal caput, caput, distal caput, corpus and cauda regions, in which the matured sperm is stored in the cauda epididymis [5]. The impairment of sperm motility is tightly associated with the male infertility, in which Ca^2+^ is a key regulator for human sperm function [6]. Calcineurin, a Ca^2+^-dependent phosphatase, is localized in both the flagellum and the post acrosomal region of the sperm head, and plays a major role in the Ca^2+^-dependent regulation of flagellar motility and acrosome exocytosis [7, 8]. Sperm-specific calcineurin consists of a catalytic subunit (PPP3CC) and a regulatory subunit (PPP3R2), and the deletion of PPP3CC/PPP3R2 resulted in male infertility due to an inflexible mid-piece of sperm in mice [9].

FK506, an immunosuppressive drug, binds to its intracellular receptors such as FK506 binding proteins (FKBPs) to inhibit the activity of calcineurin [10]. Among the 16 different human FKBPs, only FK506 binding protein 12 (FKBP12) and 12.6 (FKBP12.6) have been observed to be able to inhibit the activities of calcineurin in the presence of FK506 with a clinical relevant concentration [11]. It has been reported that FK506-induced male infertility was related to the inhibition of the activities of sperm-specific calcineurin [9]. However, the specific developmental stages of sperm affected by calcineurin and the contribution of FKBP12 or FKBP12.6 in mediating this inhibition have not been explored.

Ca^2+^ signaling has been proposed to control chemotaxis, hyperactivation and acrosomal exocytosis of sperm [12]. Intracellular Ca^2+^ stores play a central role in the regulation of intracellular Ca^2+^ ([Ca^2+^]i) and the generation of complex Ca^2+^ signals such as oscillations and waves [13]. There are at least two Ca^2+^ storage organelles in mammalian sperm, one in the acrosomal region and another in the region of the sperm neck and mid-piece [14]. Ryanodine receptors (RyRs) serve as the intracellular Ca^2+^ release channels for Ca^2+^ stores, to participate in calcium-induced calcium release, a potential mechanism for the regenerative Ca^2+^ waves during fertilization [14]. It has been documented that RyR-mediated Ca^2+^ mobilization is involved in the regulation of male germ cell maturation [15]. Proteomic analyses have confirmed that RyR2 was expressed in sperm from epididymis [16, 17], and the knockdown of RyR2 impedes progesterone-induced motility [18]. Our previous studies showed that FKBP12.6 is selectively bound to RyR2, and plays a pivotal role in the regulation of Ca^2+^ release [19–22]. Furthermore, studies indicated that calcineurin also modulated the Ca^2+^ release of RyR2 via interaction with FKBP12.6 [13]. Nevertheless, it remains unclear whether FK506 influences Ca^2+^ homeostasis by dissociating FKBP12.6 from RyR2 and/or inhibiting the activities of calcineurin through forming a complex with FKBP12.6 in sperm.

Studies indicated that mitochondria played a fundamental role in male fertility [23, 24], and calcineurin was anchored to mitochondria by spermatogenesis-Associated 33 protein to regulates sperm motility [25]. In mammalian sperm, mitochondria are confined to the mid-piece of the flagellum, a region that aligns with the area impacted by FK506 [26]. However, whether FKBP12.6 affects mitochondrial homeostasis of sperm by inhibiting calcineurin remains unclear.

In the present study, the role and the underlying mechanism of FKBP12.6 in FK506-induced male infertility have been investigated using FKBP12.6 knockout (FKBP12.6^−/−^) mice. Certainly, our study should provide an insight in elucidating the mechanisms of FK506-induced male infertility.

## Results

### FKBP12.6 deficiency alleviated FK506-induced male infertility without affecting morphology of testes and epididymides in vivo

**Fig. 1:**
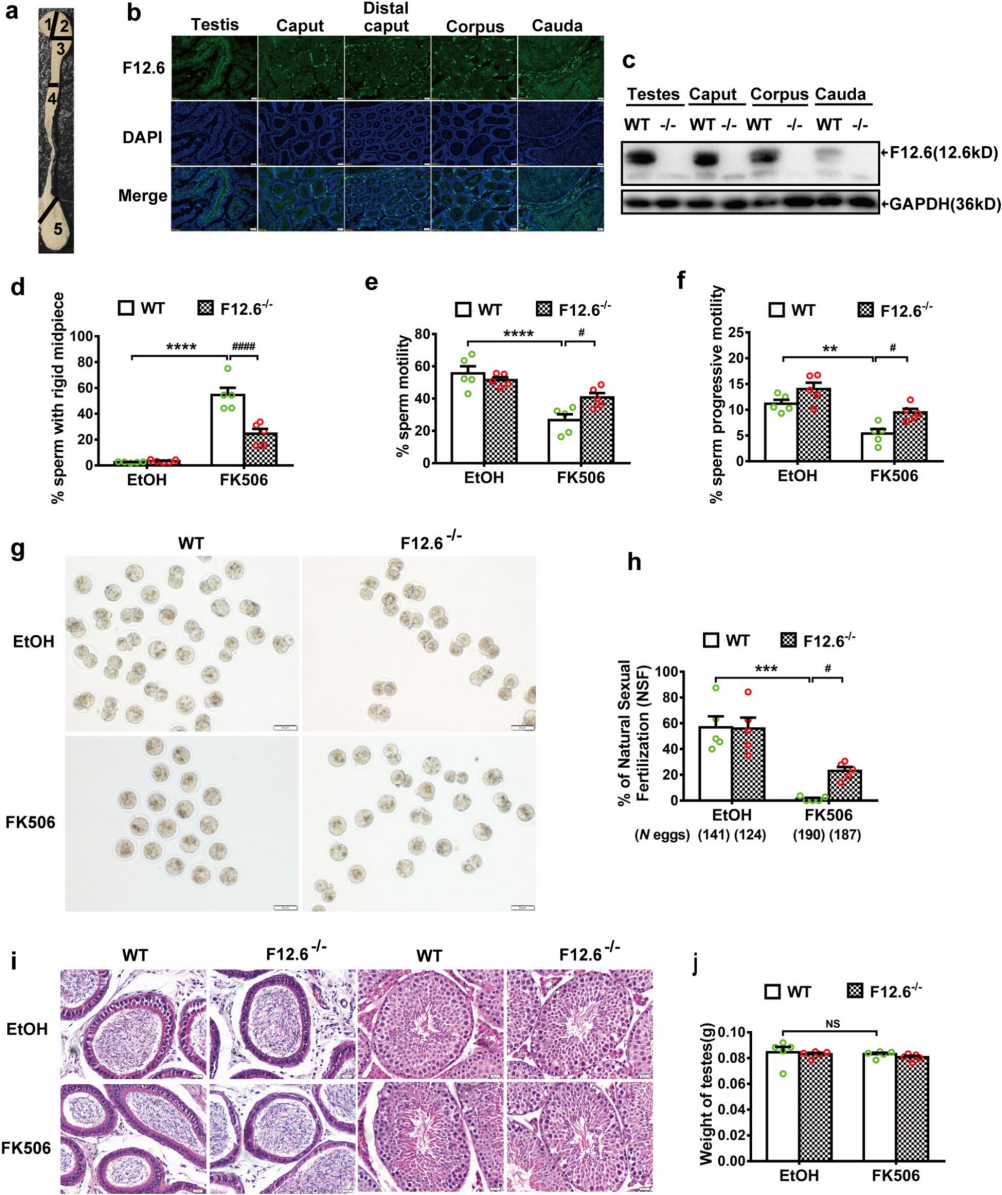
FKBP12.6 deficiency alleviated FK506-induced male infertility without affecting the morphology of the testes and epididymides in vivo. **a** The cartoon shows the five parts of sperm isolated from testes and epididymides: 1-proximal caput, 2-caput, 3-distal caput, 4-corpus, and 5-cauda. **b** The expressions of FKBP12.6 were detected by immunofluorescent staining in various parts of sperm, scale bar, 20 μm. **c** The expressions of FKBP12.6 were detected by western blot in sperm from mouse testes and the various parts of epididymides in FKBP12.6^−/−^ mice, *n* = 3. The sperm were obtained by swimming out from the cauda epididymides of male mice after injection of FK506 or an equal amount of ethanol (EtOH) for 14 days. **d** The percentages of the sperm with a rigid midpiece were randomly calculated with 200 sperm. **e–f** The total motility (**e**) and progressive motility (**f**) of sperm were analyzed by Computer Assisted Sperm Analysis. **g-h** The images (**g**) and the quantitative natural sexual fertilization (NSF, **h**) were analyzed with two-cell embryo formation, scale bar, 50 μm. **i** H&E staining of the epididymides and testes were obtained from male mice, scale bar, 20 μm. **j** The weights of testes were obtained from male mice. Data were shown as means ± SEM, *n* = 5. Significance was measured using a Sidak’s or Tukey’s post hoc test after Two-way ANOVA for multiple group comparisons. ^#^ *P* < 0.05, ** *P* < 0.01, *** *P* < 0.001, **** *P* or.^####^ *P* < 0.001, NS represented as *P* > 0.05

To investigate the role of FKBP12.6 in FK506-induced male infertility, the expression of FKBP12.6 was examined in sperm of adult male mice. The results of immunofluorescence and western blot showed that FKBP12.6 was highly expressed in the testes, and caput and corpus, but not cauda epididymides of WT mice (Fig. 1a-c), in comparison, FKBP12.6 expression was undetectable in FKBP12.6^−/−^ mice (Fig. 1c). Then WT and FKBP12.6^−/−^ adult male mice were subcutaneously injected with FK506 for 14 days, and sperm statuses were analyzed with a Computer Assisted Sperm Analysis system and the Natural Sexual Fertilization (NSF) was analyzed with cumulus intact oocytes. The results showed that FK506 notably increased the rate of sperm with a rigid mid-piece (Fig. 1d), inhibited the sperm motility (Fig. 1e) and progressive motility (Fig. 1f), and reduced the NSF rates (Fig. 1g, h) in WT mice compared with the ethanol group. Importantly, FKBP12.6 deficiency significantly reversed FK506-induced the alterations in FKBP12.6^−/−^ mice compared with WT mice. In addition, the Haematoxylin and eosin (H&E) staining showed that the morphologies of seminiferous tubules and epididymides were not altered (Fig. 1i), and the testicular weights were also not changed (Fig. 1j) in both WT and FKBP12.6^−/−^ mice treated with FK506. These results indicated that FKBP12.6 deficiency protected against FK506-induced male infertility by enhancing sperm motility without altering the morphology of the testes and epididymides in vivo.

### FKBP12.6 deficiency attenuated FK506-induced disorder of mitochondrial function accompanied with the reduction of ROS generation and the improvement of the MMP in immature sperm

**Fig. 2:**
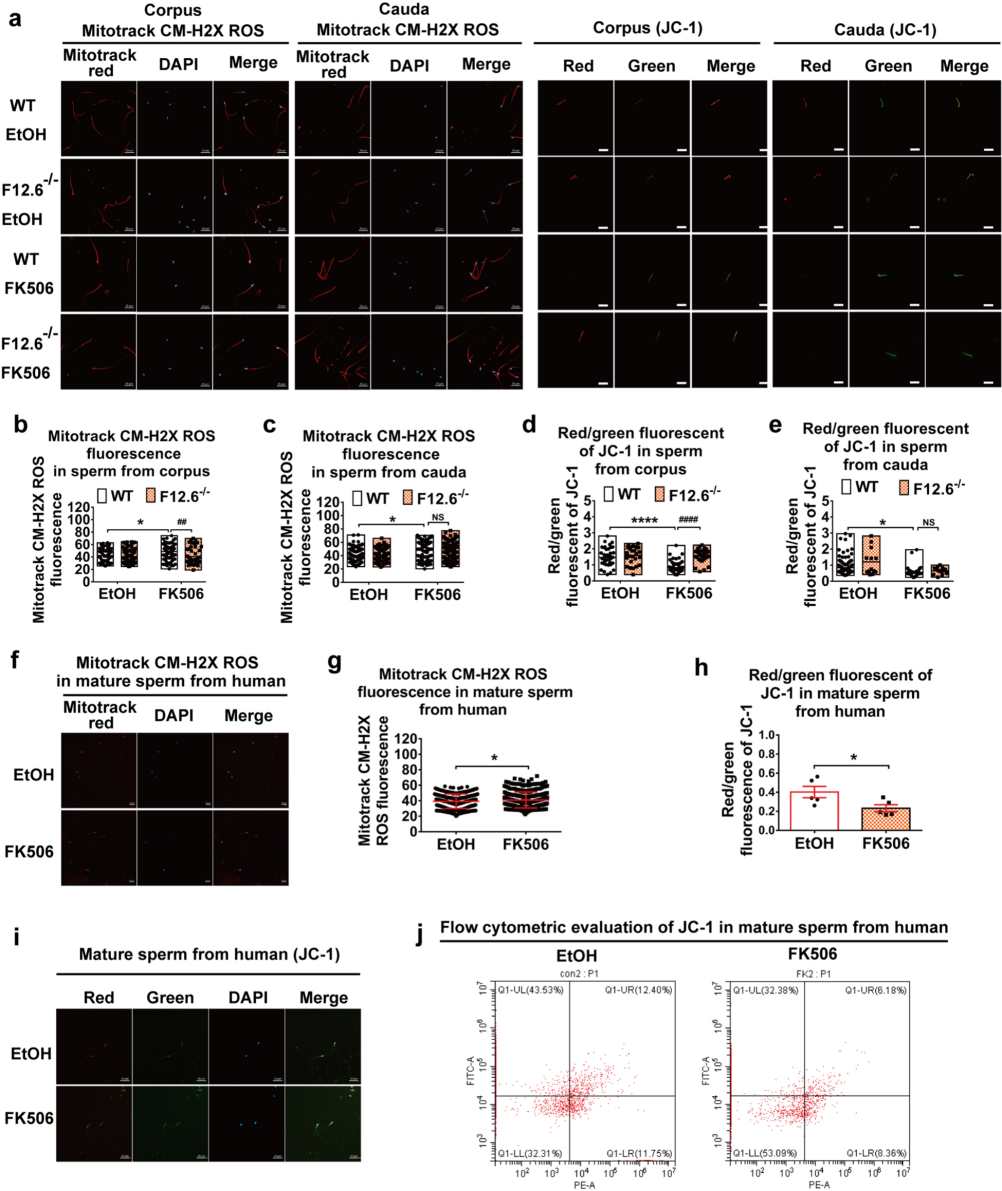
FKBP12.6 deficiency attenuated FK506-induced disorder of mitochondrial function accompanied with the reduction of ROS generation and the improvement of the MMP in immature sperm. **a** Sperm were isolated from corpus and cauda epididymides in WT and FKBP12.6^−/−^ male mice and cultured with the enriched DMEM/F12 medium containing 50 µM FK506 or 0.1% ethanol (EtOH) for 30 min. The human sperm were obtained by swimming out from human semen samples and incubated with or without FK506 for 30 min. Then, ROS generation and mitochondrial membrane potential (MMP) of sperm were measured by loading with Mitotrack CM-H2X ROS or JC-1 probes, respectively. The images of Mitotrack CM-H2X ROS and red/green fluorescence channels of JC-1 in sperm from corpus & cauda were recorded by a Zeiss LSM800 confocal laser scanning microscope with a 400 × objective, scale bar, 20 μm. **b-c** The fluorescence intensities of Mitotrack CM-H2X ROS were quantitatively analyzed in sperm from corpus (**b**) and cauda (**c**). **d-e** The ratio of red to green fluorescence intensities in JC-1 staining of sperm from the corpus epididymis (**d**) and cauda epididymis (**e**) were quantitatively analyzed. **f** The Images of Mitotrack CM-H2X ROS in human sperm were recorded by a microscope with a 200 × objective, scale bar, 20 μm. **g-i** The fluorescence intensities of Mitotrack CM-H2X ROS (**g**) and the ratios of red to green fluorescence intensities (**h**) were quantitatively analyzed by a flow cytometry in sperm, the images of red and green fluorescence channels of JC-1 (**i**) were recorded by a microscope with a 400 × objective, scale bar, 20 μm. **j** Red and green fluorescence was detected by flow cytometry using PE and FITC filter. Data were shown as means ± SEM, *n* = 3 in mice, *n* = 5 in human samples. Significance was measured using a Tukey’s post hoc test after Two-way ANOVA for multiple group comparisons in mice or unpaired t tests in humans. *P < 0.05, ^##^*P* < 0.01, ****P or.^####^*P* < 0.001, NS represented as *P* > 0.05

It has been reported that mitochondrial ROS production led to the reductions of viability, motility, and MMP in sperm [26]. To determine the effects of FK506 and FKBP12.6 on ROS generation and MMP, sperm isolated from corpus (represented immature sperm) and cauda epididymides (represented mature sperm) were loaded with Mitotrack CM-H2X ROS or JC-1 probes, respectively. The results showed that FK506 significantly increased the fluorescent intensities of Mitotrack CM-H2X ROS of sperm from corpus (Fig. 2a, b), and cauda epididymides (Fig. 2a, c), and reduced the ratios of JC-1’s red fluorescence to green fluorescence in the mid-piece of sperm in the corpus (Fig. 2a, d) and cauda epididymides (Fig. 2a, e) in WT mice. In addition, FK506 also remarkably enhanced the ROS generation in human mature sperm (Fig. 2f, g), and reduced the MMP (Fig. 2h-j) in the mid-piece of human sperm compared with the ethanol-treated group, indicating that the results from human sperm were consistent with our mouse results. Importantly, FKBP12.6 deficiency markedly reduced FK506-induced excessive ROS generation (Fig. 2a, b) and increased the ratio of JC-1’s red fluorescence to green fluorescence in sperm from the corpus (Fig. 2a, d), but not from the cauda epididymides (Fig. 2a, c, e), indicating that FKBP12.6 deficiency maintained the mitochondrial homeostasis in immature sperm.

### FKBP12.6 preferentially bound to the sperm-specific calcineurin (PPP3CC/PPP3R2) in testes

**Fig. 3:**
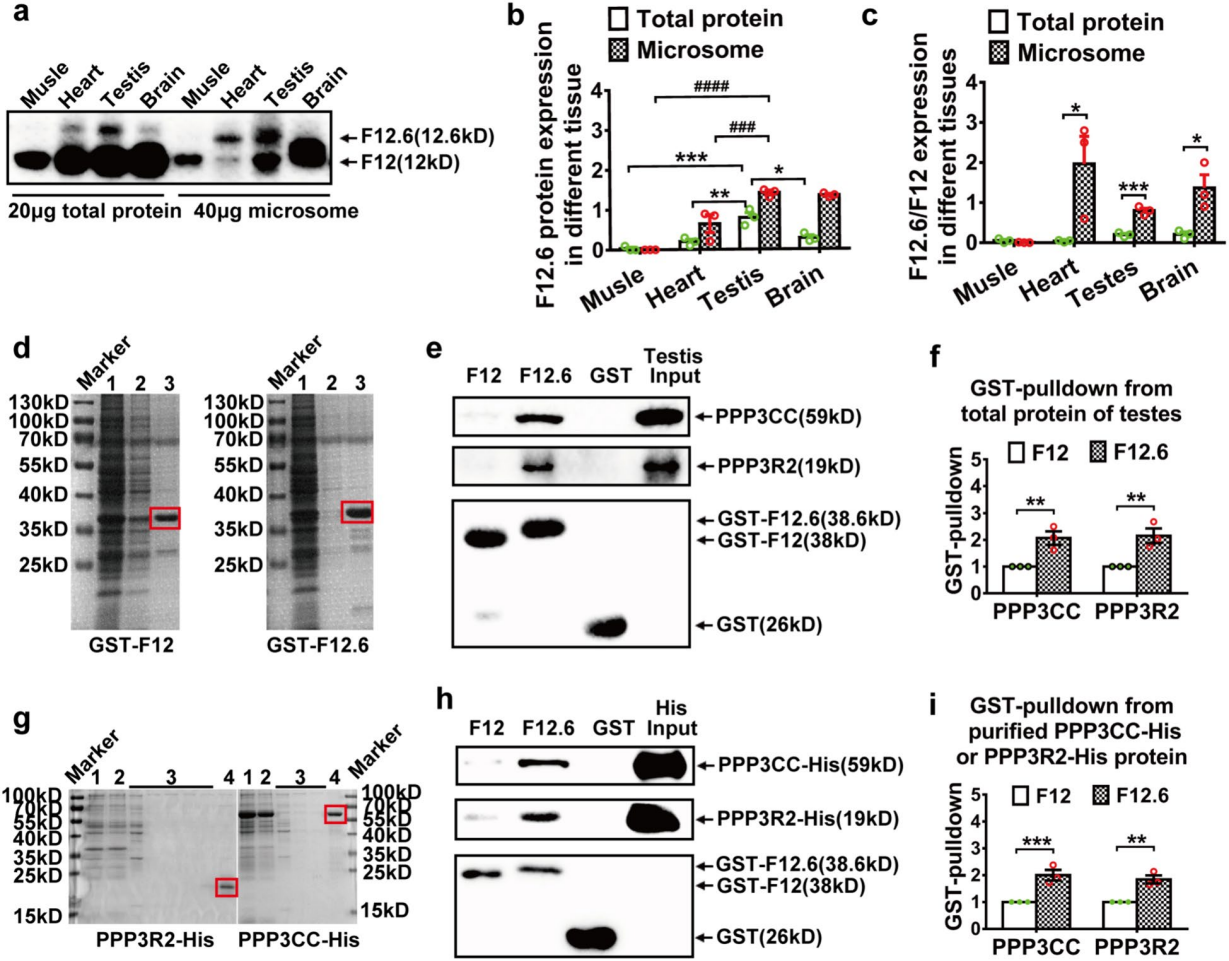
FK506-FKBP12.6 complex selectively bound to sperm-specific calcineurin (PPP3CC/PPP3R2) in testes. **a-c** The expressions of FKBP12 and FKBP12.6 protein were detected by western blot in the total protein and microsome of heart, muscle, testicular tissue and cerebral cortex (**a**), and the expressions of FKBP12.6 (**b**) and the ratio of FKBP12.6/FKBP12 expression (**c**) were quantitatively analyzed from **a**, respectively. **d** GST-tagged FKBP12.6, GST-tagged FKBP12 proteins were expressed and purified, then detected by SDS-PAGE, purified proteins were annotated with red boxes: line 1, pre-purification sample; line 2, washing liquid; line 3, elution. **e–f** The GST pull-down assay showed that GST-tagged FKBP12.6 or GST-tagged FKBP12 bound to calcineurin-catalyzed subunit PPP3CC and regulated subunit PPP3R2 in testis lysate in the presence of FK506, respectively. **g** The His-tagged PPP3CC, His-tagged PPP3R2 proteins were expressed and purified, then detected by SDS-PAGE, the purified proteins were annotated with red boxes: line 1, pre-purification sample; line 2, flow-through; line 3, washing liquid; line 4, elution. **h-i** The GST pull-down assay showed that GST-tagged FKBP12.6 or GST-tagged FKBP12 bound to His-PPP3CC and His-PPP3R2 in the presence of FK506, respectively. Data were shown as means ± SEM from three independent experiments. Significance was measured using a Sidak’s post hoc test after Two-way ANOVA or Multiple t tests for multiple group comparisons. * *P* < 0.05, ** *P* < 0.01, *** *P* or ^###^ *P* < 0.001, ^####^ *P* < 0.001

To clarify the role of FKBP12.6 in FK506-induced infertility, the expressions of FKBP12 (F12) and FKBP12.6 (F12.6) were examined in total proteins and microsomes (sarcoplasmic/endoplasmic reticulum). The results showed that FKBP12.6 was highly expressed in testes (Fig. 2a, b), and enriched in microsomes isolated from heart, testes and brain, although the expressions of FKBP12 were much higher than that of FKBP12.6 in all the tissues (Fig. 3a-c), suggesting that FKBP12.6 might play a key role in regulating Ca^2+^ release of testes. Next, to clarify the specific contribution of FKBP12.6 in FK506-induced male infertility, we prepared several tagged proteins including GST-tagged FKBP12, GST-tagged FKBP12.6, His-tagged PPP3CC, and His-tagged PPP3R2, for conducting the pull-down experiments. The results showed that the tagged proteins were successfully purified with a high purity (Fig. 2d, g). The pull-down experiments revealed that GST-FKBP12.6 specifically interacted with PPP3CC and PPP3R2 from mouse testes lysate in the presence of FK506 (Fig. 2e, f). Additionally, the purified His-tagged PPP3CC and His-tagged PPP3R2 proteins also preferentially bound to FKBP12.6 in the presence of FK506 (Fig. 3h, i). These results suggested that FKBP12.6-FK506 complex might exert a stronger inhibition to sperm-specific calcineurin (PPP3CC/PPP3R2) since the complex preferentially bound to them.

### FKBP12.6 deficiency reversed FK506-induced the elevated expression of DSCR1.1 and the reduced expression of PPP3CC/PPP3R2 in immature sperm

**Fig. 4:**
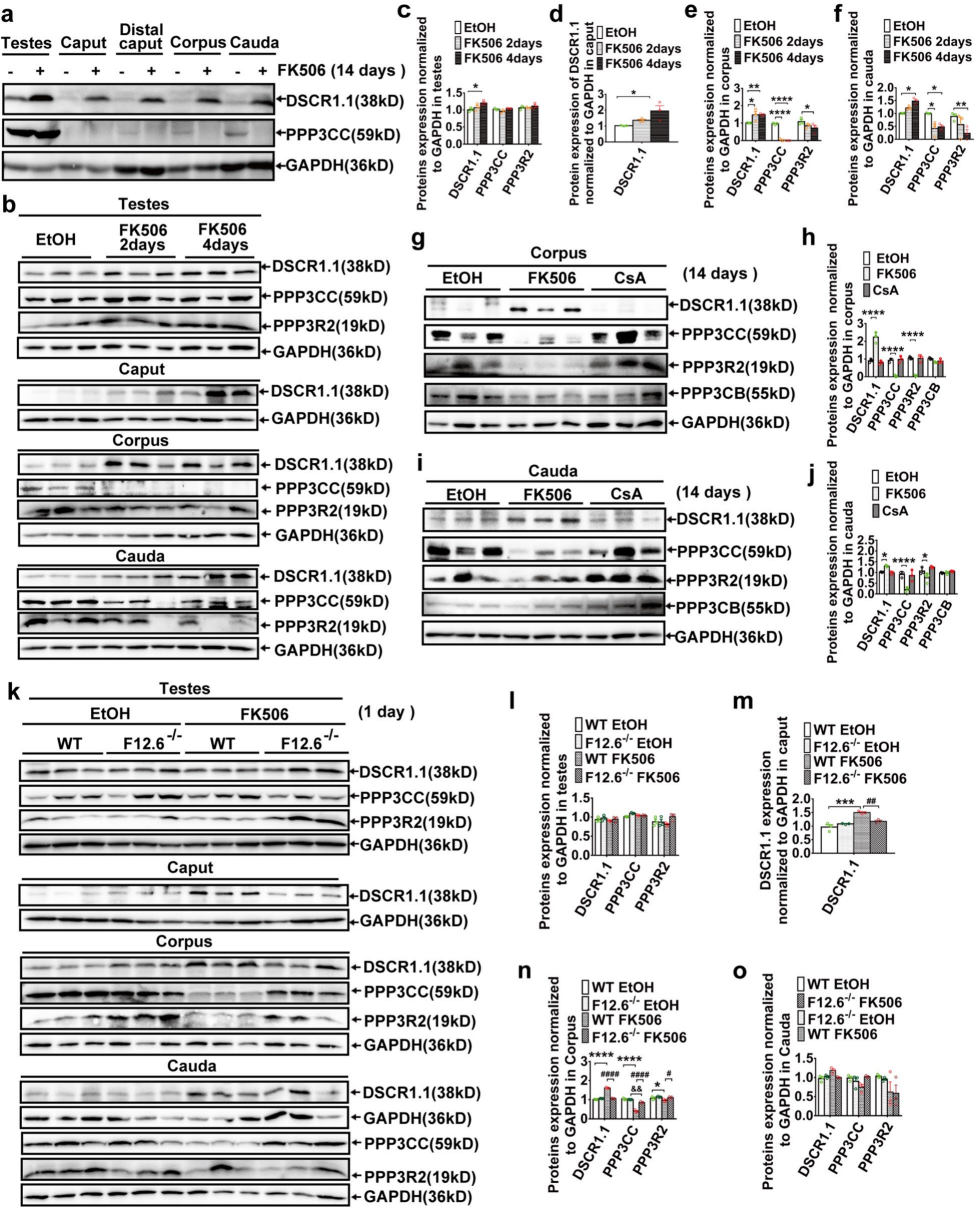
FKBP12.6 deficiency inhibited FK506-induced elevation of DSCR1.1 expression and reduction of PPP3CC and PPP3R2 expression in immature sperm. **a** The expressions of DSCR1.1 and PPP3CC were detected by western blot in testes, and in different parts of epididymides (caput, distal caput, corpus and cauda) in WT male mice injected with FK506 or ethanol for 14 days. **b-f** The expressions of DSCR1.1, PPP3CC and PPP3R2 were detected by western blot in testes, and in different parts of epididymides in WT male mice injected with FK506 or ethanol for 2 or 4 days (**b**), the expressions of these proteins were quantitatively analyzed in testes (**c**), caput (**d**), corpus (**e**) and cauda (**f**) of epididymides. **g-j** The protein expression of DSCR1.1, PPP3CC, PPP3R2 and PPP3CB were detected and quantitatively analyzed by western blot in corpus (**g, h**) and cauda (**i, j**) of epididymides in WT male mice injected with FK506 or CsA or an equal amount of ethanol for 14 days. **k–o** The expressions of DSCR1.1, PPP3CC and PPP3R2 were detected and quantitatively analyzed by western blot in testes (**l**), and in caput (**m**), corpus (**n**) and cauda (**o**) epididymides in WT and FKBP12.6^−/−^ male mice injected with FK506 or ethanol for one day (**k**). The data were shown as means ± SEM, *n* = 3. Significance was measured using a Sidak’s or Tukey’s post hoc test after Two-way or One-way ANOVA for multiple group comparisons. * *P* or ^#^ *P* < 0.05, ** *P* or ^##^ *P* or ^&&^ *P* < 0.01, *** *P* < 0.001, **** *P* or.^####^ *P* < 0.0001

To further clarify the effects of FK506 and FKBP12.6 on the inhibition of sperm-specific calcineurin, the expressions of Down syndrome critical region 1.1 (DSCR1.1) (an inhibitor of calcineurin [27]), PPP3CC and PPP3R2 were analyzed following FK506 administration in vivo. Our results showed that the expression of DSCR1.1 was significantly increased in the testes and epididymides, while the expression of PPP3CC was remarkably decreased in the distal caput, corpus, and cauda epididymides of WT mice treated with FK506 for 14 days (Fig. 4a). Similar results were observed in various developmental stages of sperm in mice treated with FK506 for 2 or 4 days (Fig. 4b-f). In addition, the mRNA expressions of FKBP12 and FKBP12.6 were observed in testes and epididymides (Fig. S1a-c), suggesting that FK506 might inhibit the activities of calcineurin in sperm throughout the entire sperm maturation process after testicular spermatogenesis. In addition, our results also showed that FK506, but not cyclosporin A (CsA), increased the expression of DSCR1.1, and reduced the expressions of PPP3CC and PPP3R2 in both corpus epididymides (Fig. 4g-h and Fig. S2a, c) and cauda epididymides (Fig. 4i-j and Fig. S2b, d) after 14 or 7 days FK506 administration. However, considering the transcription of sperm in the epididymides has ceased, our RT- PCR results showed that the mRNA expressions of DSCR1.1, PPP3CC, and PPP3R2 (Fig. S2e) were moderately increased following FK506 treatment in testes of mice, suggesting that the decreased protein expressions of PPP3CC and PPP3R2 induced by FK506 might be due to degradation.

Next, to determine the roles of FKBP12.6 in FK506-induced the elevation of DSCR1.1 expressions and the decrease of the expressions of PPP3CC and PPP3R2 in different developmental stages of sperm, we performed FK506 administration for one day, a very short time in vivo to minimize the impact of sperm migration in the testes and epididymides. Our western blot results showed that FK506 significantly increased the expressions of DSCR1.1 in sperm from the caput (Fig. 4k, m) and corpus epididymides (Fig. 4k, n), and markedly reduced the expressions of PPP3CC and PPP3R2 in sperm from the corpus epididymides (Fig. 4k, n), but not sperm from cauda epididymides (Fig. 4k, o) of WT mice, suggesting that FK506 might preferentially inhibit the function of immature sperm. Importantly, FKBP12.6 deficiency remarkably prevented the increased expressions of DSCR1.1 the decreased expressions of PPP3CC and PPP3R2 (Fig. 4k, m, n) in immature sperm of mice treated with FK506. These results indicated that FKBP12.6 deficiency restored calcineurin function of the immature sperm in FK506-induced male infertility through reducing the expression of DSCR1.1, and retaining the expressions of PPP3CC/PPP3R2.

### FKBP12.6 deficiency reversed FK506-induced abnormality of Ca^2+^ release through restoring calcineurin-mediated dephosphorylation at S2808 and S2814 sites of RyR2

**Fig. 5:**
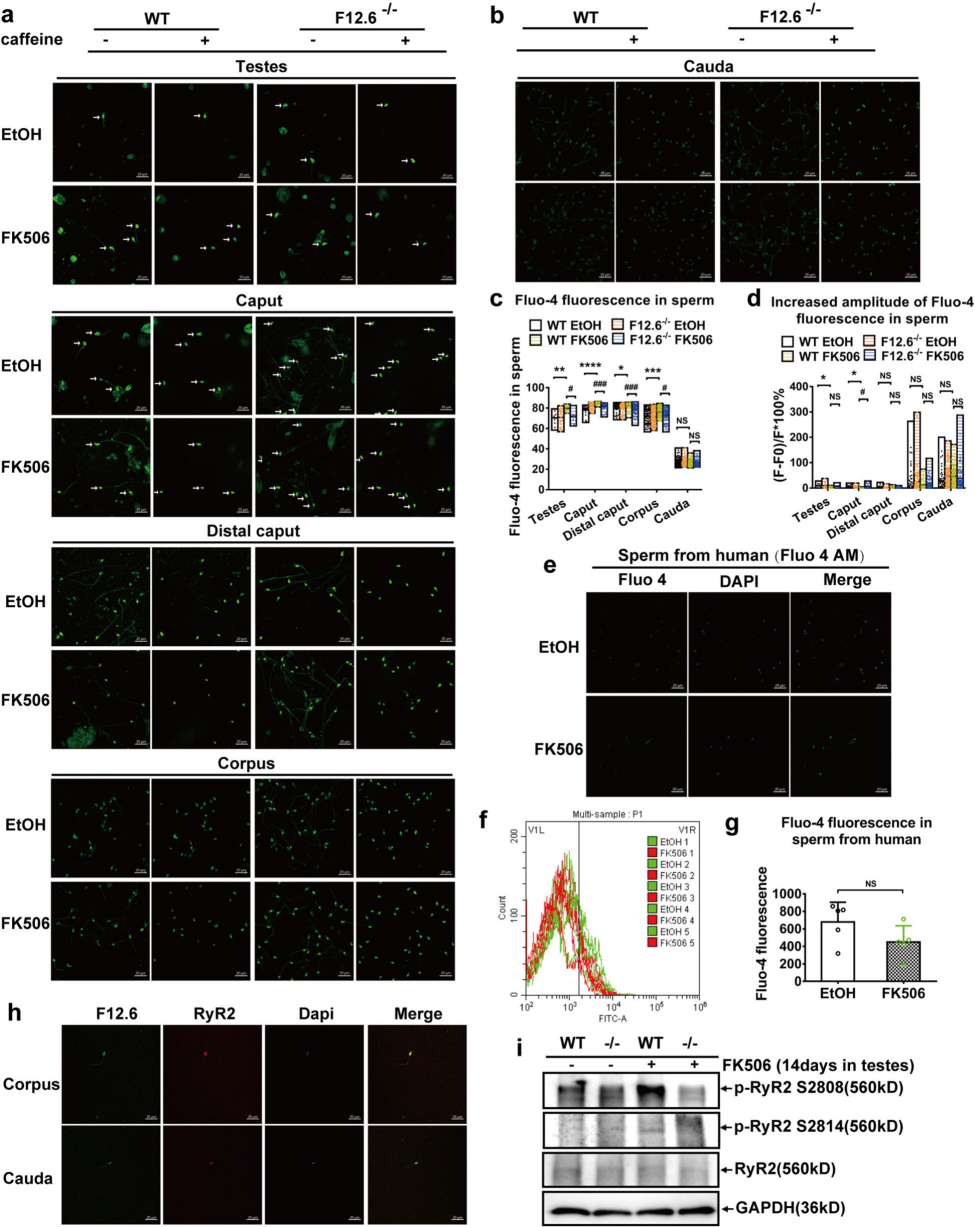
FKBP12.6 deficiency reversed FK506-induced abnormality of Ca^2+^ release through restoring calcineurin-mediated dephosphorylation at S2808 and S2814 sites of RyR2. Sperm were isolated from testes and caput, distal caput, corpus epididymides in mice, then sperm were treated with 50 μM FK506 or 0.1% ethanol for 30 min, and were loaded with Fluo-4 AM for detecting [Ca^2+^]i, fluorescence was recorded before and after addition of 50 μl (1:1 dilution) solutions containing 10 mM caffeine. **a-b** The images were taken using a Zeiss LSM800 confocal laser scanning microscope with a 400 × objective in testes, and in caput, distal caput, corpus (**a**), and cauda of epididymides (**b**), respectively, scale bar, 20 μm. The white arrows indicated sperm from testes and caput epididymides. **c-d** The fluorescent intensities of FK506-induced [Ca^2+^]i (**c**) and caffeine-induced Ca^2+^ release from Ca^2+^ stores (**d**) were quantitatively analyzed in testes, and in caput, distal caput, corpus and cauda of epididymides, respectively. **e–g** Human sperm were obtained by swimming out from the human semen samples and incubated with or without FK506 for 30 min, and then the sperm were loaded with Fluo-4 AM for detecting [Ca^2+^]i. The images were taken using a Zeiss LSM800 confocal laser scanning microscope with a 400 × objective, scale bar, 20 μm (**e**), and the fluorescence was recorded and analyzed by flow cytometry (**f, g**). **h** Immunofluorescence colocalization staining was used to detect the colocalization expressions of FKBP12.6 and RyR2 in sperm form corpus and cauda epididymides of WT male mice, The images were taken using a Zeiss LSM800 confocal laser scanning microscope with a 400 × objective, scale bar, 20 μm. **i** The expressions of total RyR2 protein, phosphorylated RyR2 at site of Ser2808 and Ser2814 were detected by western blot in testes from WT and FKBP12.6 deficiency mice injected with FK506 or ethanol for 14 days. Data were shown as means ± SEM, *n* = 3 in mice, *n* = 5 in human. Significance was measured using a Tukey’s post hoc test after Two-way ANOVA for multiple group comparisons in mice or unpaired t tests in humans. * *P* or ^#^ *P* < 0.05, ** *P* or ^##^ *P* < 0.01, *** *P* or ^###^ *P* < 0.001, **** *P* or.^####^ *P* < 0.0001, NS represented as *P* > 0.05

To clarify the role of FKBP12.6 in Ca^2+^ release of sperm, the intracellular Ca^2+^ release was examined using Fluo-4 at different developmental stages of sperm. The results showed that FK506 significantly increased the concentration of intracellular Ca^2+^ ([Ca^2+^]i) (Fig. 5a, c, Fig. S3a-c), and reduced caffeine-induced Ca^2+^ release from intracellular Ca^2+^ stores in the immature sperm (sperm from testes and caput, distal caput, corpus epididymides) (Fig. 5a, c), but not in the mature sperm (sperm from cauda epididymides) from WT (Fig. 5b-d) compared with ethanol group. In addition, consistent with our mouse results, the [Ca^2+^]i of human mature sperm was also not affected by FK506 (Fig. 5e-g). Importantly, the disruption of FKBP12.6 significantly reversed FK506-induced the abnormal Ca^2+^ release in immature sperm (Fig. 5a-d).

To elucidate the mechanism of FKBP12.6 in regulating Ca^2+^ release in sperm, the distributions of three RyRs at different stages of sperm maturation in mice were detected by RT-PCR, immunofluorescence and western blot. Our results showed that RyR2 was expressed in the innermost layer of the seminiferous tubules in the testis, similar to the expression of FKBP12/12.6 (Fig. S4), FKBP12.6 and RyR2 colocalization in sperm from corpus and cauda epididymides (Fig. 5h), and FK506 markedly increased the phosphorylation at the Ser2808 (p-RyR2 S2808) and Ser2814 (p-RyR2 S2808) sites of RyR2 in WT mice, but not in FKBP12.6^−/−^ mice (Fig. 5i). These results indicated that FKBP12.6 deficiency markedly reversed FK506-induced abnormality of Ca^2+^ release through restoring calcineurin-mediated dephosphorylation at S2808 and S2814 sites of RyR2.

### FKBP12.6 deficiency decreased FK506-induced hyperphosphorylation at Ser637 of Drp1 through retaining the activities of calcineurin in immature sperm

**Fig. 6:**
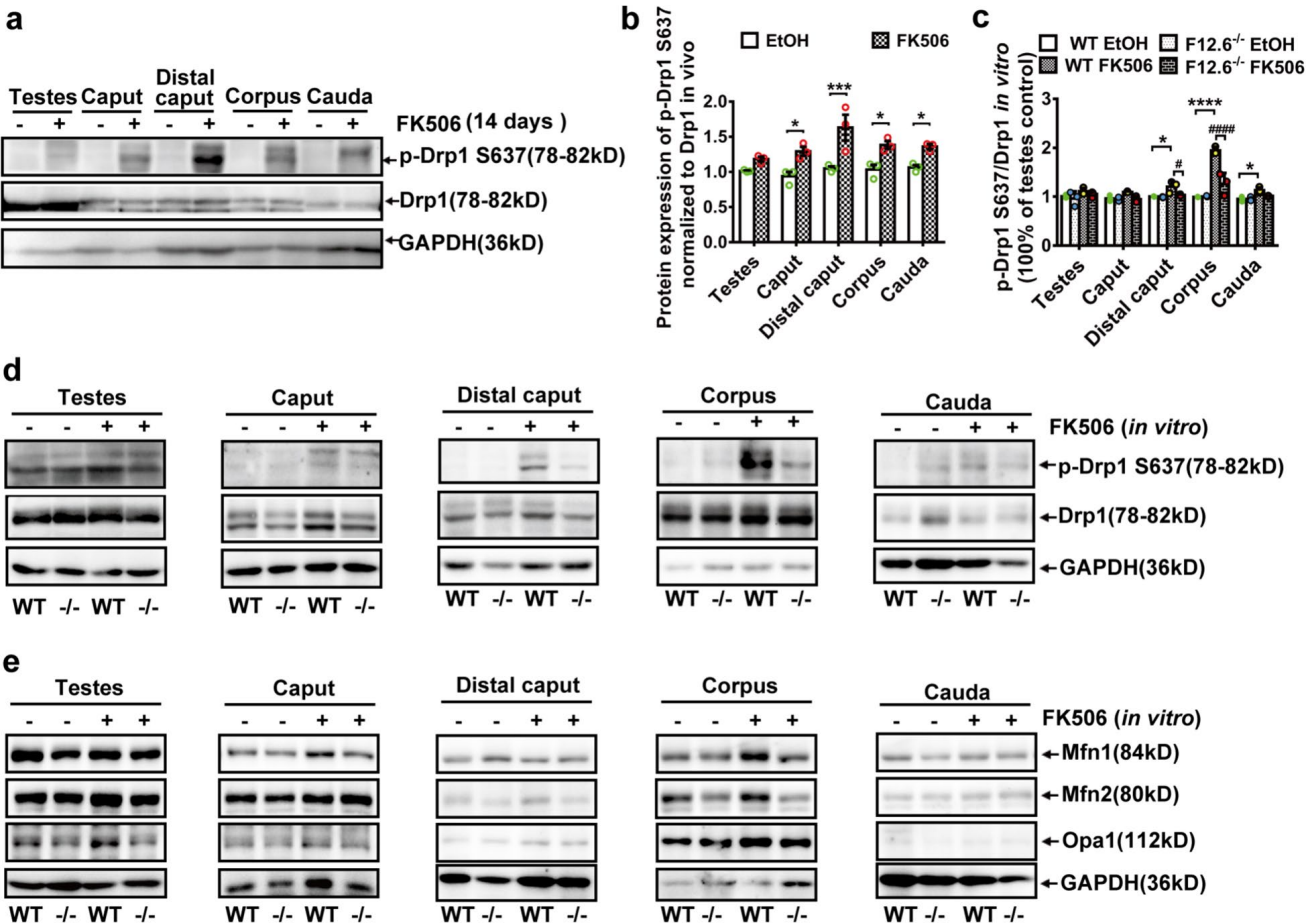
FKBP12.6 deficiency reduced FK506-induced the hyperphosphorylation of Drp1 at S637 in immature sperm. **a-b** The expressions of total and phosphorylated Drp1 at site of Ser637 were detected by western blot in testes, and in different parts of epididymides (caput, distal caput, corpus and cauda) isolated from WT male mice injected with FK506 or ethanol for 14 days. Data were shown as means ± SEM, *n* = 3. **c-d** For in vitro studies, tissues from 6 numbers of WT or FKBP12.6^−/−^ mice were mixed together to collect enough sperm cells, then sperm were isolated from testes, and caput, distal caput, corpus, cauda epididymides of WT and FKBP12.6^−/−^ male mice and cultured in the enriched DMEM/F12 medium containing 50 µM FK506 or 0.1% ethanol for 2 h, and the images of the expressions of total and phosphorylated Drp1 at site of Ser637 were detected and quantitatively analyzed by western blot in testes, and in caput, distal caput, corpus, cauda epididymides. **e** The expressions of mitochondrial fusion proteins such as Mfn1, Mfn2 and Opa1 were detected by western blot in the different developmental stages of sperm. Data were shown as means ± SEM from three independent experiments. Significance was measured using a Sidak’s or Tukey’s post hoc test after Two-way ANOVA for multiple group comparisons. * *P* or ^#^ *P* < 0.05, *** *P* < 0.001, **** *P* or.^####^ *P* < 0.0001

To clarify the roles of FK506 in the mitochondria, the expression of the phosphorylation at Ser637 of Drp1 were detected. Our results showed that the expression of the phosphorylated Drp1 at Ser637 site (p-Drp1 S637) was conspicuously increased in different developmental stages of sperm after 14 days of FK506 administration (Fig. 6a, b). To further determine the roles of FKBP12.6 in the mitochondria of the immature and mature sperm, different developmental stages of sperm were treated with FK506 in vitro. Our western blot results showed that FK506 significantly increased the phosphorylation level of Drp1 Ser637 in sperm isolated from WT mice (Fig. 6c, d), and FKBP12.6 deficiency remarkably reversed FK506-induced alterations of sperm isolated from the distal caput and corpus epididymides, but not in cauda epididymides (Fig. 6c, d) in FKBP12.6^−/−^ mice compared with WT mice. Moreover, the expressions of mitochondrial fusion proteins such as Mfn1, Mfn2 and Opa1 were not affected by FK506 in sperm isolated from WT or FKBP12.6^−/−^ mice (Fig. 6e). These results indicated that FKBP12.6 deficiency reduced FK506-induced hyperphosphorylation of Drp1 Ser637 in immature sperm, which might also contribute to their protection on FK506-induced male infertility.

### FKBP12.6 deficiency restored FK506-induced the reduction of the sperm acrosome exocytosis by inhibiting Snap25 downregulation

**Fig. 7:**
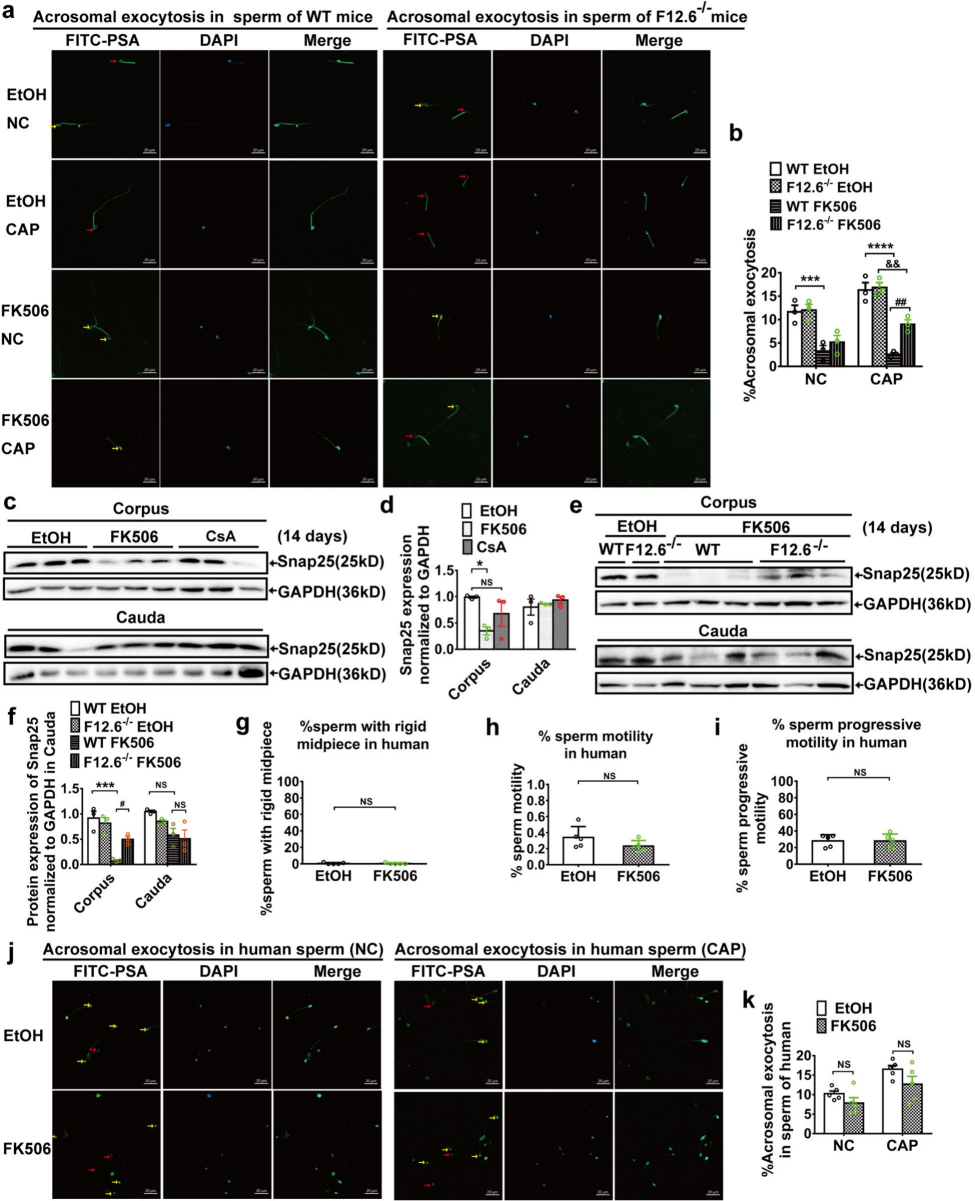
FKBP12.6 deficiency retained sperm acrosome exocytosis maturation by inhibiting Snap25 downregulation. **a-b** After consecutive 14 days FK506 or equal amount of ethanol injection, acrosomal exocytosis in non-capacitating (NC), and CAP sperm induced by 10 µM A23187 were imaged using a Zeiss LSM800 confocal laser scanning microscope with a 400 × objective, scale bar, 20 μm, the yellow arrow points to the sperm with a complete acrosome, the red arrow points to the sperm that had undergone acrosome reaction (**a**), and the acrosomal exocytosis was quantified in non-capacitating (NC) and CAP sperm (**b**). **c-d** The expression of Snap25 in corpus and cauda epididymides in WT male mice were detected and quantitatively analyzed by western blot. **e–f** The expression of Snap25 in corpus and cauda epididymides in WT and FKBP12.6^−/−^ male mice were detected and quantitatively analyzed by western blot. Data were shown as means ± SEM, *n* = 3. **g-i** The human mature sperm were obtained by swimming out from the human semen samples and incubated with or without FK506 for 2 h, and the percentages of the sperm with a rigid midpiece were randomly calculated with 200 sperm (**g**), and the total sperm motility (**h**) and progressive motility (**i**) in sperm were analyzed by Computer Assisted Sperm Analysis. **j-k** Acrosomal exocytosis in non-capacitating (NC), and CAP sperm induced by 15 µM A23187 were imaged and quantitatively analyzed with human sperm. Data were shown as means ± SEM, *n* = 5. Significance was measured, Tukey’s post hoc test after One-way or Two-way ANOVA for multiple group comparisons were used in (**b**,** d**,** e**), unpaired t tests were used in (**g-i**), multiple t tests were used in (**k**) * *P* or.^#^ *P* < 0.05, *** *P* < 0.001, NS represented as *P* > 0.05

To determine the roles of FK506 and FKBP12.6 in sperm, acrosome exocytosis and the expression of synaptosome-associated protein of 25 kDa (Snap25) were analyzed. Our results showed that FK506 significantly reduced the rate of sperm undergoing the acrosome reaction (assessed via acrosome exocytosis staining with PSA-FITC) (Fig. 7a, b). In addition, the expression of Snap25 in immature sperm was downregulated by FK506 (Fig. 7c-f), but not CsA (Fig. 7c, d) in WT mice, whereas FKBP12.6 deficiency protected against the reduction of the acrosome reaction rate (Fig. 7a, b) and the reduced expression of Snap25 (Fig. 7e, f) induced by 14 days FK506 treatment in vivo. Furthermore, our results also showed that FK506 did not affect the rate of sperm with a rigid mid-piece, sperm motility, progressive motility (Fig. 7g-i), and the rate of the acrosome reaction (Fig. 7j, k) of human mature sperm in vitro. These results indicated that FK506 influenced the maturation process of sperm such as acrosome exocytosis in immature sperm, but not mature sperm. Therefore, FKBP12.6 deficiency also preserved the capacity for the maturation of acrosome exocytosis function in sperm by inhibiting the downregulation of Snap25.

## Discussion

Studies have demonstrated that inhibition of calcineurin—mediated by FK506 and cyclosporine A (CsA) through their respective binding to FKBP12/12.6 and cyclophilins—attenuates sperm motility [9, 28, 29]. Clinical studies indicated that FK506 led to a decline in the fertilization capacity of male renal transplant recipients [30, 31]. In addition, male patients with Down Syndrome usually suffered from infertility accompanied with the overexpression of DSCR1 [27, 32, 33]. DSCR1 is a calcineurin inhibitor that is likely to directly contact the enzyme active site [34, 35]. In the present study, we observed that FK506 notably elevated the expression of DSCR1.1, and reduced the expressions of sperm-specific calcineurin (PPP3CC/PPP3R2) in sperm. Interestingly, this phenomenon had not been observed during CsA treatment, indicating that FK506-induced the elevated expression of DSCR1.1, and the decreased expressions of PPP3CC and PPP3R2 were mediated by FKBP12.6 and/or FKBP12. Furthermore, the inhibition of FK506 on calcineurin was amplified by this specific mechanism, providing a new explanation for the fact that the inhibitory effect of FK506 on calcineurin is much stronger than that of CsA.

In general, sperm maturation in the epididymis is almost exclusively controlled by post-translational modification of their intrinsic protein complement or transfer of exosomes released from the epididymal epithelium [36], suggested that the upregulation of DSCR1.1 was due to post-translational modification or synthesized by the epididymal epithelium and then incorporated into the sperm cells. We are not clear whether the degradation of PPP3CC/PPP3R2 were via the ubiquitin–proteasome pathway or other protein degradation pathways. In somatic cells, FK506 enhanced the degradation of calcineurin by interfering with the interaction between PPP3CA and calcineurin B [37]. We speculated that the FK506-induced the degradation of PPP3CC/PPP3R2 might be related to the interference locally. Given the fact that the FK506-induced the upregulation of DSCR1.1 and degradation of PPP3CC/PPP3R2 were more pronounced in WT mice compared to FKBP12.6^−/−^ mice, suggesting that FKBP12.6 might mediate the FK506-induced these alterations by suppressing calcineurin activities in mice.

Meistrich et al. reported that sperm maturation from the testis to the cauda epididymis requires about 4–10 days [38]. This temporal framework aligns with the observation that abnormalities in cauda epididymal sperm emerged within 4 to 5 days following treatment with CsA or FK506 [9], suggesting that sperm-specific calcineurin might affect sperm motility in the early stage of sperm maturation in the testis. To clarify which developmental stages of sperm were affected by FK506, we have examined the effects of FK506 administration on mouse fertility at different time points of day 1, 2, 4, 7, and 14, and 14 days of FK506 treatment covered the required time for sperm to transit the epididymis. Our data demonstrated that FKBP12.6 deficiency significantly inhibited infertility induced by 14 days FK506 treatment, and remarkably reversed FK506-induced the increased expressions of DSCR1.1 and the declined expression of PPP3CC and PPP3R2 in immature sperm even for a very short time of FK506 administration, indicating that FKBP12.6 mediated the FK506-induced damage to the entire sperm maturation process in mice.

Intracellular Ca^2+^ serves as a main regulator of sperm motility, and the intracellular Ca^2+^ content was reduced in the caudal sperm of PPP3R2 knockout mice [29]. FK506 increased [Ca^2+^]i via the mobilization of intracellular Ca^2+^ stores since this effect was accompanied with a decreased intraluminal Ca^2+^ content [39]. Our results showed that FKBP12.6 deficiency significantly attenuated FK506-induced the increase of Ca^2+^ release in sperm, and increased the sperm intracellular calcium store content in immature sperm, suggesting that FK506 might also lead to Ca^2+^ leaking from RyR2 and lower intracellular calcium store content in sperm, most likely through combining to FKBP12.6 in the entire sperm maturation process after testicular spermatogenesis. Besides, we also observed that, as the same as during capacitation [40], only a subpopulation of mouse sperm displayed a rapid increase in intracellular calcium induced by caffeine. Protein kinase A and CaMKII phosphorylated RyR2 at Ser2808 [41], and at Ser2808, Ser2814 [42], respectively. Phosphatase 1 appears to be the main phosphatase for dephosphorylating Ser2808 and Ser2814 of RyR2 [43]. Inhibition of calcineurin would lead to RyR2 hyperphosphorylation through hyperphosphorylation of I-1, a selective and potent phosphatase 1 inhibitor [44]. In our study, FKBP12.6 deficiency significantly attenuated FK506-induced Ca^2+^ release through reducing the hyperphosphorylation of RyR2 at Ser2808 and Ser2814 sites, suggesting that FKBP12.6 might also play a key role in regulating calcium release through inhibiting the dephosphorylated activity of calcineurin.

The dynamin-related protein 1 (Drp1) is dephosphorylated by calcineurin at serine 637 (Ser637) [45, 46]. We demonstrated that FK506 significantly enhanced the phosphorylation of Drp1 at Ser637, which could mediate the inhibition of mitochondrial division, and the disturbance of mitochondrial fusion and division induces overloaded mitochondrial reactive oxygen species (ROS) production [45], whereas the excessive ROS reduced sperm viability, motility, and mitochondrial membrane potential (MMP) [26]. Meanwhile, FKBP12.6 deficiency remarkably reduced FK506-induced ROS generation and increased the MMP via suppressing the hyperphosphorylation of Drp1 Ser637, but it failed to exert a protective effect on mature sperm in mice. Our results indicated that the inhibition of FKBP12.6 may not benefit to the mature sperm following FK506 administration. The protective effects of FKBP12.6 deficiency on immature sperm may be related to the following two aspects. On one hand, the inhibitory effect of FK506 on immature sperm is involved in the acrosome reaction, calcium homeostasis, and mitochondrial dysfunction, whereas its effect on mature sperm is solely limited to the disruption of mitochondrial homeostasis. On the other hand, the expression level of FKBP12.6 in mature sperm is much lower than that in immature sperm, suggesting that the effect of FKBP12.6 on mature sperm was limited.

Miyata et al. demonstrated that PPP3CC null sperm could pass through the cumulus cell layers and bind to the zona pellucida, but failed to fertilize cumulus-free ZP intact oocytes [9]. Acrosome exocytosis happens after zona binding [47], Snap25 is a plasma membrane Q (containing glutamate)-SNARE for Ca^2+^-dependent secretory vesicle-plasma membrane fusion [48], and it is essential for acrosome exocytosis after intraacrosomal Ca^2+^ efflux [49, 50]. Many studies reported that the expression of Snap25 protein was reduced in Down Syndrome [51, 52], moreover, the reduced exocytosis levels were observed in mice with DSCR1 overexpression [53], indicating that FK506 might also affect the ability of maturation of acrosome exocytosis function in sperm through regulating the expression of Snap25. Indeed, our results showed that the expression of Snap25 was reduced by FK506, but not CsA, and FKBP12.6 deficiency also retained the ability of the maturation of acrosome exocytosis function in sperm through inhibiting degradation of Snap25 in immature sperm.

In the present study, we did provide strong evidence to our conclusion that FKBP12.6 is a potential therapeutic target for FK506-induced male infertility: firstly, FKBP12.6 deficiency significantly protected male mice from FK506-induced infertility, second, FKBP12.6 was highly expressed in testis, caput and corpus epididymides, but not cauda epididymides, third, FKBP12.6 deficiency remarkably attenuated FK506-induced the disorder of calcium release and mitochondrial function in immature sperm, and fourth, FK506-FKBP12.6 complex selectively bound to and inhibited the sperm-specific calcineurin activities in testes.

However, some limitations also exist in our present study. Currently, we did not obtain the bleeding data from FKBP12.6^−/−^ mice following long-term FK506 treatment due to the poor mental status in mice. Additionally, a small molecule specifically inhibiting FKBP12.6 is not available, therefore, we failed to validate the role of FKBP12.6 in FK506-induced male infertility in vitro. Furthermore, we did not completely exclude the role of FKBP12 in FK506-induced infertility, since the complex of FK506-FKBP12 was also able to pull down some specific PPP3CC/PPP3R2 in the testes in our study, suggesting that the high level of intracellular FKBP12 might overcome the low affinity to PPP3CC/PPP3R2 in testes to make a certain contribution to FK506-induced calcineurin inhibition. FKBP12.6 plays a primary and indispensable role although a potential minor contribution of FKBP12 cannot be entirely ruled out and warrants further validation.

Although literature has demonstrated that intracytoplasmic sperm injection (ICSI) enabled the majority of male patients to achieve reproductive success, approximately one-third of organ transplant recipients still fail to produce offspring following ICSI [54]. Furthermore, for those patients who have undergone transplantation with immunosuppressants, the risk of preeclampsia is elevated in the offspring conceived via ICSI [55]. In the present study, we demonstrated that FKBP12.6 did not affect mouse fertility outcomes, sperm motility and acrosome reaction function of mature sperm, or participate in baseline regulation of sperm-specific calcineurin, maintenance of mitochondrial homeostasis and modulation of Ca^2^⁺ signaling under physiological conditions. Therefore, targeting FKBP12.6 may offer high specificity and avoid interference with normal reproductive physiology, suggesting promising safety characteristics in clinical application for FK506-induced male infertility.

## Materials and methods

### Experimental animals

FKBP12.6^−/−^ mice were generated in our previous study. Briefly, the FKBP12.6 gene was disrupted in embryonic stem cells by homologous recombination using a targeting vector in which exon 3, coding for the binding sites for RyR2, FK506 and calcineurin, was replaced by a neomycin-resistance (neor) cassette. FKBP12.6^−/−^ mice grow at normal rates without problematical fertile [19]. In the present study, FKBP12.6^−/−^ mice with 129 s background were back crossed with C57BL/6 J mice for more than 10 generations to generate the deficiency mice with C57BL/6 J genetic background. The male mice at the age of 3–4 months were used and housed in a 12:12-h light/dark cycle (lighting changes occurring at 06:00 and 18:00 h) with food and sterile water plus libitum.

### Sperm motility analysis

Mice treated with or without FK506 were euthanized and their cauda epididymides were then removed. Sperm were isolated as described by wang et al. [56]. Human sperm were purified by swim-up and treated with or without FK506 in vitro. Sperm motility was analyzed with a Computer Assisted Sperm Analysis system (WLJY-9000, WeiLi Co., Ltd., Beijing, China). Three parameters (rigid midpiece, total sperm motility and progressive motility) were recorded. A minimum of 200 sperm were counted for each assay.

### Natural Sexual Fertilization (NSF)

C57BL/6 J WT female mice with ages of 4–6 weeks were super ovulated with 5 IU of pregnant mare serum gonadotropin (PMSG, NSHF, Ningbo, China). 48 h later, they were administered with 5 IU of post-human chorionic gonadotropin (hCG, NSHF, Ningbo, China). These WT female mice were coupled with male mice treated with FK506 or ethanol. Copulation was confirmed by checking for vaginal plugs in the next morning. Then, female mice were euthanized and their oocytes were collected from the cumulus-oocyte complex in KSOM containing hyaluronidase (MR-106 and H4272, Sigma-Aldrich, Merck, Melbourne, Australia) and carefully moved into fresh KSOM medium and incubated for 4 h at 37 °C. The percentage of two-cell embryos was considered as NSF rate.

### Western blot analysis

The proteins were separated by electrophoresis on 6% to 15% SDS-PAGE gel and transferred to PVDF membrane (Millipore, MA, USA). Expressions of proteins were detected following standard protocols and normalized to the reference GAPDH. The antibodies used in this study were listed in supplementary table S3.

### FK506 or Cyclosporine A (CsA) administration

In vivo FK506 or CsA administration: FK506 (Cat# MB1232, Meilunbio, Dalian, China) or CsA (Cat# HY-B0579, MCE, New Jersey, USA) were dissolved in ethanol (20 mg or 200 mg/ml) and diluted with Cremophor EL (Sigma-Aldrich). The diluted FK506 or CsA solutions were injected into the male mice based on body weight (2 µl/g, 8 mg or 80 mg/kg) every day with subcutaneous injection for 1, 2, 4, 7 or 14 days. Ethanol diluted with Cremophor EL was used as a control (EtOH) [9].

In vitro FK506 administration: FK506 was dissolved in ethanol (50 mM) and diluted with the medium at a final concentration of 50 µM for FK506 groups, and 0.1% ethanol was used as control groups.

### Preparation of mouse sperm

Mice were euthanized, testes and epididymides were then removed and shifted into PBS, separated from fat and connective tissue. The coatings of testes were removed, the epididymides were dissected into four anatomical regions corresponding to the caput, distal caput, corpus and cauda [57]. Sperm from testes and epididymides were isolated and cultured in enriched DMEM/F12 medium as described by Chang YF et al. [58].

### Preparation of human sperm

Human semen samples were obtained from the First Affiliated Hospital of Nanchang University Fertility and Reproductive Medicine Center. The clinic confirmed that all samples met normal semen parameters according to the World Health Organization. Then sperm were purified by swim-up in noncommercial HTF as described by Shweta Bhagwat et al. [59]. Each sample was then equally divided into FK506 group and ethanol control group. After 30 min or 2 h, each sample was collected by centrifuge at 150 × g for 3 min at 4℃.

### Haematoxylin and eosin (H&E) staining

Testes and epididymides were collected and preserved by fixation in 4% paraformaldehyde for a full 24 h. The tissue blocks were sectioned into 5 μm slices and treated with H&E following standard protocols.

### In vitro pull-down assay

Mouse PPP3CC and PPP3R2 were cloned into His-labeled PET-28a through EcoR1, Xho1 and BamH1, Xho1 sites respectively. The primer pairs of musPPP3CC and musPPP3R2 were listed in the Supplementary Table S1. pGEX-4 T-3 (GST), pGEX-4 T-3-FKBP12 (GST-FKBP12), pGEX-4 T-3-FKBP12.6 (GST-FKBP12.6), His-labeled PET-28a-PPP3CC (His-PPP3CC) and PET-28a-PPP3R2 (His-PPP3R2) plasmids were transfected into BL21 (DE3) *E. coli* to express the fusion proteins. Then the bacteria were sonically lysed, and the supernatant was obtained by centrifugation and purified by ProteinIso® GST Resin (Cat# DP201, TransGen Biotech, Beijing, China), the inclusion bodies obtained by centrifugation were dialyzed and refolded by urea gradient, and then purified by Ni–NTA 6FF Sefinose (TM) Resin Kit (BBI) according to the manufacturer's instructions. 1 mg purified GST or GST-FKBP12.6 or GST-FKBP12 fusion proteins were mixed with 200 μg whole lysis of testes or 15 μg purified His-PPP3CC and 15 μg His-PPP3R2; and then they were co-incubated with FK506 at a final concentration of 10 μM. Protein complexes were precipitated with 150 μL ProteinIso® GST Resin beads overnight at 4 °C, then these beads were washed with PBS three times. The bound proteins were subjected to immunoblotting with indicated antibodies.

### Real-time quantitative PCR (RT-PCR) analysis

Real-time quantitative PCR (RT-PCR) analysis was performed as described previously [60]. The primer pairs of GAPDH, FKBP12, FKBP12.6, RyR1, RyR2, RyR3, DSCR1.1, PPP3CC and PPP3R2 were listed in the Supplementary Table S2.

### Immunofluorescence staining

Testes and epididymides were collected and preserved by fixation in 4% paraformaldehyde for a full 24 h. Then they were dehydrated with a sucrose gradient phosphate solution, embedded with Optimal Cutting Temperature compound (OTC), and sectioned into 20 μm slices on a cryostat microtome. Then slices were washed with PBS and blocked with 10% goat serum, and were then subjected to overnight immunostaining at 4 °C with monoclonal antibodies targeting FKBP12, FKBP12.6, RyR1 and RyR2, followed by incubation with Alexa Fluor-tagged or Ifluor conjugated secondary antibodies for one hour. The final images were obtained using fluorescence microscopy or confocal *microscope*.

### Preparation of tissue microsome

Mouse skeletal muscle, heart, brain and testis were prepared as described previously [61]. Briefly, tissues were homogenizing with 8 volume of HB buffer (5 mM Imidazole, pH 7.4, 0.3 M sucrose with cOmplete™ protease and phosSTOP™ inhibitor cocktail tablets) and entrifuged at 20,000 × g for 10 min at 4℃, supernatant for each sample were centrifuged at 120,000 × g for 1 h at 4℃. The final pellet (microsome) was resuspend with HB buffer, and the samples were frozen with liquid nitrogen and stored at −80℃.

### Mitotrack CM-H2X ROS and JC-1 staining

Sperm from different groups were stained with 200 nM MitoTracker® probe (Cat# HWYS29946, HWRK, Beijing, China.) or 5 μM JC-1 (Cat# HWYS85049, HWRK, Beijing, China.) for 20 min at 37 °C in a 5% CO2 incubator shielded from light, 10 μM CCCP (Cat# HWYS85757, HWRK, Beijing, China) was added as a positive control for 30 min before JC-1 staining. Then the sperm were washed and re-suspended with PBS. 15 μl of sperm were placed on a warmed microscope slide and the resulting red or green fluorescence was recorded immediately using a confocal microscope. For human sperm, fluorescence of JC-1 was also recorded using a flow cytometer (Cyto Flex, Beckman Coulter, CA 92821, USA) with two fluorescent detectors (PE and FITC).

### Measurement of intracellular free Ca^2+^ in sperm

Changes in intracellular free Ca^2+^ concentrations were measured in non-capacitated sperm. sperm were maintained in enriched DMEM/F12 medium after being released out from the testes, caput, distal caput, corpus and cauda epididymides. Then sperm with or without FK506 were stained with 5 μM Fluo-4 (Fluo-4, AM, cell permeant Invitrogen F14201) and 0.05% pluronic F-127 (Cat# 9003–11-6, Sigma-Aldrich, Merck, Melbourne, Australia) for 30 min at room temperature in the dark, sperm were washed and re-suspended with no Ca^2+^ PBS. 15 μl washed sperm were loaded on polylysine (Cat# P2658, Sigma-Aldrich, Merck, Melbourne, Australia) coated coverslips of glass bottom cell culture dishes (Φ, 1.5 cm, Nest Biotechnology Co., Ltd.), and were allowed to attach for 20 min [62]. For human sperm, fluorescence was recorded using a flow cytometer (Cyto Flex, Beckman Coulter, CA 92821, USA) equipped with a 488-nm laser, the images were also taken using a confocal *microscope*. For mouse sperm, fluorescence was recorded in both before and after the addition of 15 μl solutions containing 10 mM caffeine to assess the capacity of the Ca^2+^ store [13]. The images were taken using a confocal *microscope*. Time series scanning was used at 512 × 512 pixels per second for 30 cycles. Intracellular [Ca^2+^] was presented as (F − F0)/F0 ratios after background subtraction, where F − F0 is the increased change in the fluorescence signal intensity, and F0 is the baseline as calculated by averaging 5 frames before caffeine stimulus application [40].

### Acrosomal exocytosis (FITC-PSA)

FKBP12.6^−/−^ and WT mice were euthanized after EtOH or FK506 treatment for 14 days, and sperm were isolated from the cauda epididymides. Human sperm were treated with EtOH or FK506 for 2 h in vitro. Sperm capacitation and acrosomal exocytosis were induced as described by Shweta Bhagwat et al. [59]. Subsequently, the samples were permeabilized with Triton-X100 diluted with PBS (0.3% v: v) for 5 min, the sperm were centrifuged and washed with PBS, then incubated with Pisum sativum agglutinin (PSA)-FITC conjugate (final concentration, 0.0375 mg/mL diluted with PBS) for 20 min at room temperature in the dark [63], and 15 μl of each sample was smeared onto a glass slide. Slides were air-dried and coverslips were mounted with DAPI dye liquor at room temperature. The images were taken using confocal microscope. A 488 nm laser line was used for excitation. Sperm with bright, uniformly stained acrosomes were counted as intact, and sperm with equatorial or no staining in the acrosomal region were counted as reacted [59].

### Statistical analysis

Fluorescence intensity of images were processed and analyzed with Image J 1.52a. Regions of interest were drawn on each sperm for fluorescence quantification. For the statistical analysis in this study, GraphPad Prism 7.0 was used. The results were presented as mean ± SEM or line at mean. Unpaired t test was used for two group comparisons, Multiple t tests for multiple group comparisons, Sidak’s post hoc test was used after Two-way or One-way analysis of variance (ANOVA) for multiple group comparisons that compare each cell mean with the other cell mean in that row, whereas Tukey’s post hoc test was used after Two-way or One-way ANOVA for multiple group comparisons that compare cell means regardless of rows and columns. Differences with a *P*-value of < 0.05 were considered statistically significant.

## Supplementary Information

Supplementary Material 1.

## Data Availability

All data supporting the findings of this study are available within the paper and its Supplementary Information.

## Abbreviations

ANOVA: Analysis of variance
[Ca^2+^]i: Intracellular Ca^2+^
CsA: Cyclosporin A
Drp1: Dynamin-related protein 1
DSCR1.1: Down syndrome critical region 1.1
EtOH: Ethanol
FKBP12: FK506 binding protein 12
FKBP12.6: F12.6, FK506 Binding Protein 12.6;
FKBPs: FK506 binding proteins
H&E: Haematoxylin and eosin
MMP: Mitochondrial membrane potential
NSF: Natural Sexual Fertilization
p-Drp1 S637: Phosphorylation of Drp1 at Ser637 site
PPP3R2: Protein Phosphatase 3, Regulatory Subunit Gamma
PPP3CC: Protein Phosphatase 3, Catalytic Subunit Gamma
PPP3CC/PPP3R2: Sperm-specific calcineurin
ROS: Reactive oxygen species
RT-PCR: Real-time quantitative Polymerase Chain Reaction
RyR1: Ryanodine receptor 1
p-RyR2 S2808: phosphorylation of RyR2 at the Ser2808 sites
p-RyR2 S2814: phosphorylation of RyR2 at the Ser2814 sites
RyR2: Ryanodine receptor 2
RyR3: Ryanodine receptor 3
RyRs: Ryanodine receptors
Snap25: Synaptosome-associated protein of 25 kDa
WT: Wild Type
