# Supplementary material for: FK506 binding protein 12.6-mediated inhibition of sperm-specific calcineurin is essential for FK506-induced male infertility by disturbing the homeostasis of calcium and mitochondria
Source: Mol Biomed. 2025 Dec 22;6:149. doi: 10.1186/s43556-025-00391-3 (PMC12722626; doi:10.1186/s43556-025-00391-3)
Supplement: Supplementary file 1 — Supplementary Material 1. [file 43556_2025_391_MOESM1_ESM.docx]

**Supplementary data**

**FK506 Binding Protein 12.6-Mediated Inhibition of Sperm-Specific Calcineurin Is Essential for FK506-Induced Male Infertility by Disturbing the Homeostasis of Calcium and Mitochondria**

Yun-Fei Xiao^1,2*^, Shi-Fen Yang^1,2,3^, Shi-Ang Huang^1,2,3^, Zhi-Xiong Zeng^1^, Li-Na Gong^1^, Lin Xie^1,4^, Ling-Fang Wang^1,2^, Xiao-Hui Guan^1,2^, Yi-Song Qian^1,2^, Mei-Xiu Jiang^1,2^, Ke-Yu Deng^1,2,3,*^ and Hong-Bo Xin^1,2,3,*^

^1^National Engineering Research Center for Bioengineering Drugs and the Technologies, Institute of Translational Medicine, Jiangxi Medical College, Nanchang University; ^2^Jiangxi Province Key Laboratory of Bioengineering Drugs, Institute of Translational Medicine, ^3^School of Pharmacy, Jiangxi Medical College, Nanchang University, Nanchang 330031, P.R. China; ^4^Jiangxi Provincial People's Hospital, the First Affiliated Hospital of Nanchang Medical College, Nanchang 330031, P.R. China.

^*^Correspondence to: Hong-Bo Xin, email: [xinhb@ncu.edu.cn](mailto:xinhb@ncu.edu.cn); Yun-Fei Xiao, email: [xyf.84@163.com;](mailto:xyf.84@163.com;) or Ke-Yu Deng, email: [dky@ncu.edu.cn](mailto:dky@ncu.edu.cn).

*Running Title: FKBP12.6 deficiency protects against male infertility*

This file includes:

Supplementary Figures and legends S1, S2, S3, S4

Supplementary Table S1, Table S2 and Table S3

**Supplementary Figures and Legends**


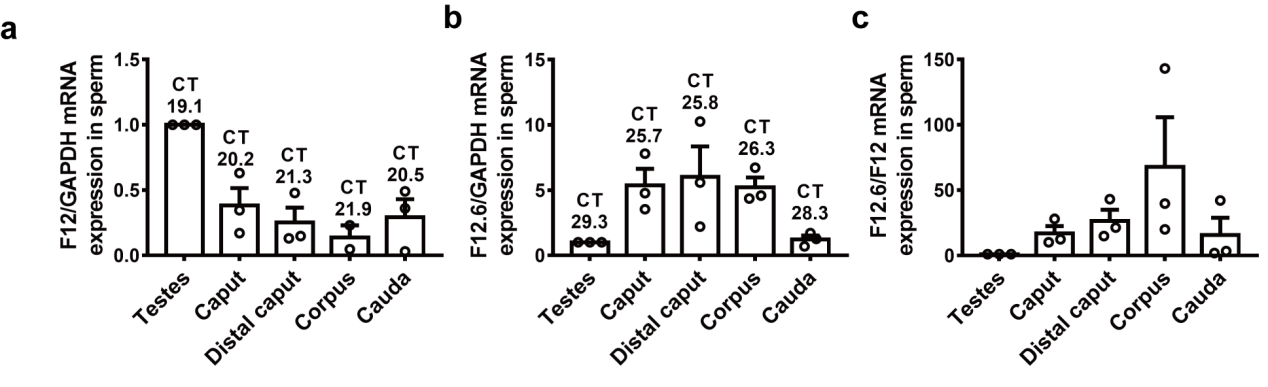


**Figure S1 The expressions of FKBP12 and FKBP12.6 in testis and epididymis**

**a-c.** Quantitative analysis of the mRNA expressions of FKBP12 and FKBP12.6 were detected by RT-PCR in testes and different parts of epididymides (caput, distal caput, corpus and cauda) isolated from WT male mice. Data were shown as means ± SEM, n = 3 per group.

**
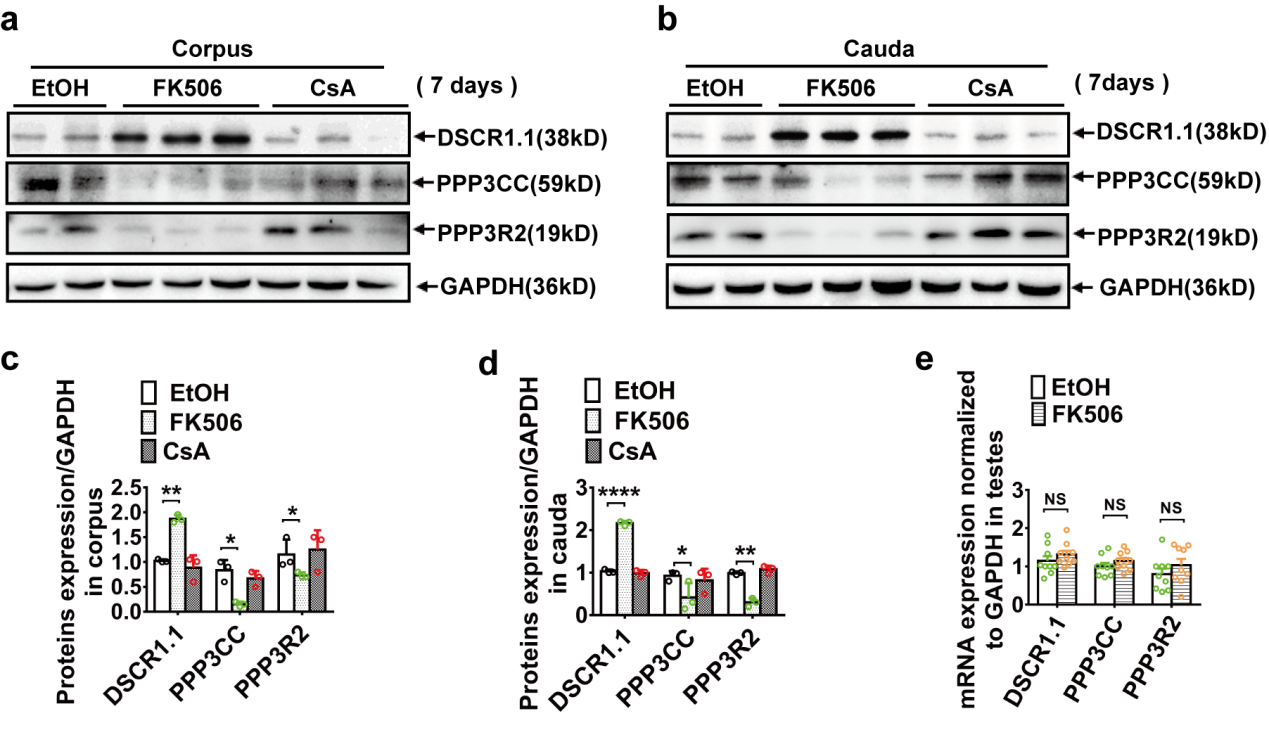
**

**Figure S2 Effects of FK506 and CsA on the expressions of DSCR1.1, PPP3CC and PPP3R2**

**a-d**. The protein expressions of DSCR1.1, PPP3CC and PPP3R2 were detected and quantitatively analyzed by western blot in corpus (**a, c**) and cauda (**b, d**) of epididymides in WT male mice injected with FK506 or CsA or an equal amount of ethanol for 7 days *in vivo.* **e**. The mRNA expressions of DSCR1.1, PPP3CC and PPP3R2 were detected by RT-PCR in testes of WT injected with FK506 or an equal amount of ethanol for 14 days. Data were shown as means ± SEM, n = 3 in **a-d**, and n = 9 in **e**. Significance was measured using a Tukey’s post hoc test after Two-way ANOVA for multiple group comparisons. **P* < 0.05, ***P* < 0.01, ****P* < 0.001, *****P* < 0.0001.

**
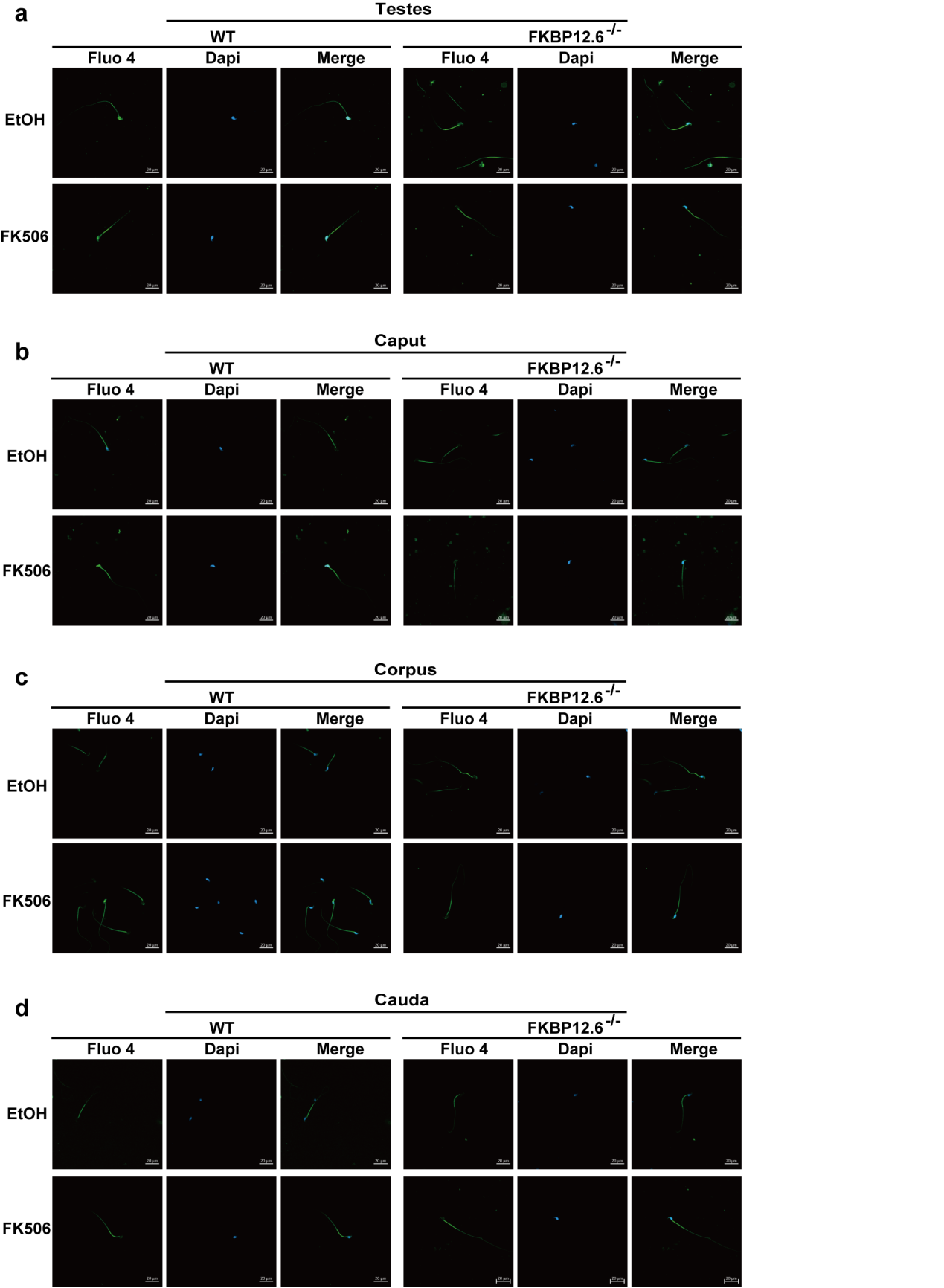
**

**Figure S3 Effects of FKBP12.6 deficiency on FK506-induced the abnormality of intracellular Ca^2+^ release in sperms of WT or FKBP12.6^-/-^ mice**

**a-d**. The sperm from testes and epididymides were isolated from WT and FKBP12.6^-/-^ male mice, then sperm were treated with 50 μM FK506 or 0.1% ethanol for 30 minutes, and were loaded with Fluo-4 AM for detecting [Ca^2+^]i. Images of testes (**a**), caput (**b**), corpus (**c**), and cauda (**d**) epididymides were taken using a Zeiss LSM800 confocal laser scanning microscope with a 400 × objective, scale bar, 20 μm.

**
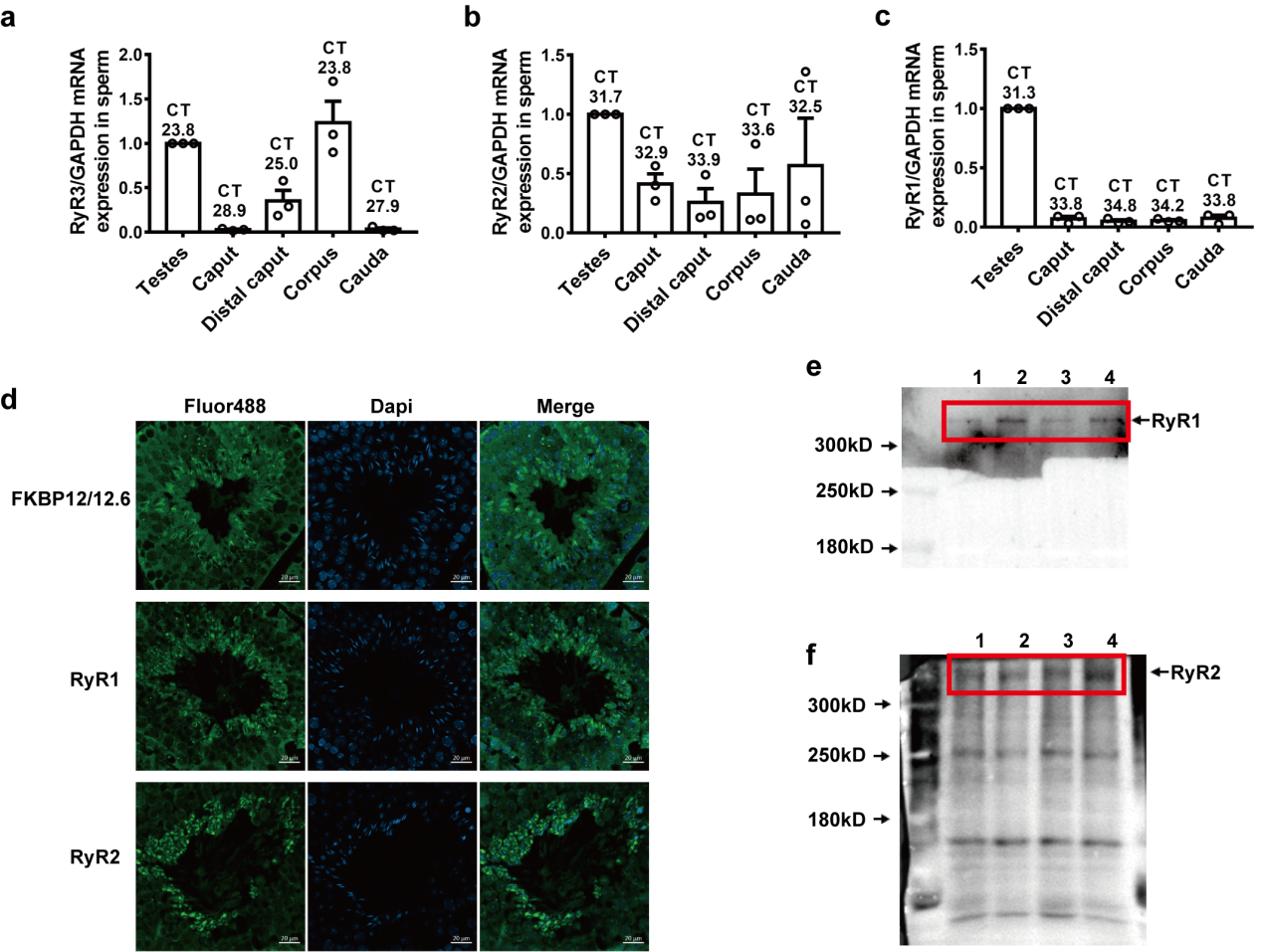
**

**Figure S4 The expressions of RyR2 and RyR1 in testes and epididymides**

**a-c**. Sperm were isolated from the testes and various sections of the epididymides in wild-type male mice. The mRNA expressions of RyR3 **(a)**, RyR2 **(b)** and RyR1 **(c)** were detected by RT-PCR. **d.** Immunofluorescent staining was used to detect the protein expressions of FKBP12/12.6, RyR1 and RyR2 in the testes of WT male mice (scale bar, 20 μm). **e-f**. Western blot was also conducted to detect RyR1**(e)** and RyR2 **(f)** expressions in testicular microsomes, with the bands annotated in red boxes: lane 1, WT EtOH group, lane 2, FKBP12.6^-/-^ EtOH group, lane 3, WT FK506 group, lane 4, FKBP12.6^-/-^ FK506 group. Data were shown as means ± SEM, n = 3 per group.

**Supplementary** **Table S1. Primers for construction of recombinant DNA (FP: forward primer, RP: reverse primer)**

| Targets | Primers |
| --- | --- |
| His-PPP3CC | 5’-GGAATTCGAGGAGACCATGTCCGTGAG-3’ (FP)  5’-CCGCTCGAGGAGTCATTACAGGGCTTTCT-3’ (RP) |
| His-PPP3R2 | 5’-CGGGATCCATGGGAAATGAGGCCAGCTAC-3’ (FP)  5’-CCGCTCGAGTGAGAAAGAAAGGCAAAGAT-3’ (RP) |

**Supplementary Table S2. Primers for RT-PCR (FP: forward primer, RP: reverse primer)**

| Targets | Primers |
| --- | --- |
| GAPDH | 5’-AGCCAAAAGGGTCATCATCT-3’ (FP)  5’-GGGGCCATCCACAGTCTTCT-3’(RP) |
| FKBP12 | 5’-CTCTCGGGACAGAAACAAGC-3’ (FP)  5’-AGAGTGGCATGTGGTGGGA-3’ (RP) |
| FKBP12.6 | 5’-CCAGGAGACGGAAGGACAT-3’ (FP)  5’-CAAAGATGAGGGTGGCATTG-3’ (RP) |
| RyR1 | 5’-CCTTGGCTTCAGCCTTCTG-3’ (FP)  5’-TCTGGGAGAGACACCTGTTGT-3’ (RP) |
| RyR2 | 5’-ATGGCTTTAAGGCACAGCG-3’ (FP)  5’-CAGAGCCCGAATCATCCAGC-3’ (RP) |
| RyR3 | 5’-ATCGCTGAACTCCTGGGTTTG-3’ (FP)  5’-CCATGAGGGGTCGTGTCAAAG-3’ (RP) |
| DSCR1.1 | 5’-GACTGGAGCTTCATCGACTGC-3’ (FP)  5’-CCCAGGAACTCGGTCTTGT-3’ (RP) |
| PPP3CC | 5’-GAAGAAGGAGCTACTACAGGTCG-3’ (FP)  5’-CCTTGAGGGTCAGCACATTC-3’ (RP) |
| PPP3R2 | 5’-GAGATAAGAAGGCTGGGTAA-3’ (FP)  5’-TCTGTGTCGAAGATGTCGAT-3’ (RP) |

**Supplementary table S3:Antibodies**

| **Antibodies** | **Source** | **Catalog Number and Identifier** |
| --- | --- | --- |
| PP2B-Agamma(M-17)/PPP3CC | Santa Cruz Biotechnology | Cat#sc-6122, RRID:AB_2168326 |
| [PP2B-B2(C-19)/PPP3R2](https://www.antibodyregistry.org/AB_2268623) | [Santa Cruz Biotechnology](https://www.antibodyregistry.org/AB_2268623) | Cat#sc-6120, RRID:AB_2268623 |
| [DSCR 1(G-2)/MCIP1](https://www.antibodyregistry.org/AB_11149927) | Santa Cruz Biotechnology | Cat#sc-377507, RRID:AB_11149927 |
| [FKBP12 Polyclonal Antibody](https://www.antibodyregistry.org/AB_2102731) | Thermo Fisher Scientific | Cat#PA1-026A, RRID:AB_2102731 |
| [FKBP12.6 Polyclonal Antibody](https://www.antibodyregistry.org/AB_2102731) | R and D Systems | Cat#AF4174, RRID:AB_2246838 |
| FKBP12.6 (H-8) | [Santa Cruz Biotechnology](https://www.antibodyregistry.org/AB_2268623) | Cat#sc-376135, RRID:AB_10991116 |
| [RyR1(D4E1)Rabbit mAb](https://www.antibodyregistry.org/AB_2797637) | Cell Signaling Technology | Cat#8153, RRID:AB_2797637 |
| [Anti-Ryanodine Receptor 2](https://www.antibodyregistry.org/AB_570808) | Millipore | Cat#AB9080, RRID:AB_570808 |
| [Ryanodine Receptor(phospho S2808)antibody](https://www.antibodyregistry.org/AB_946327) | Abcam | Cat#ab59225, RRID:AB_946327 |
| [Phospho-RYR2(Ser2814)Antibody](https://www.antibodyregistry.org/AB_2845317) | Affinity Biosciences | Cat#AF2303, RRID:AB_2845317 |
| [DRP1(D8H5)Rabbit mAb](https://www.antibodyregistry.org/AB_11178938) | Cell Signaling Technology | Cat#5391, RRID:AB_11178938 |
| [Phospho-DRP1(Ser637)(D3A4)Rabbit mAb](https://www.antibodyregistry.org/AB_10971640) | Cell Signaling Technology | Cat#6319, RRID:AB_10971640 |
| [Recombinant Anti-Mitofusin 1 antibody[EPR21953-74]](https://www.antibodyregistry.org/AB_2941083) | Abcam | ab221661, RRID:AB_2941083 |
| [Mitofusin 2 antibody[NIAR164]](https://www.antibodyregistry.org/AB_10999860) | Abcam | Cat#ab124773, RRID:AB_10999860 |
| [Recombinant Anti-OPA1 antibody[EPR11057(B)]](https://www.antibodyregistry.org/AB_2864313) | Abcam | Cat#ab157457, RRID:AB_2864313 |
| Monoclonal Mouse Anti-Glyceraldehyde-3-Phosphate Dehydrogease(GAPDH) | Kangchen Biotech | Cat#KC-5G4, RRID:AB_2493106 |
| Goat anti-Rabbit IgG(H+L)Secondary Antibody, HRP | Invitrogen™ | Cat#31460, RRID:AB_228341 |
| [Goat anti-Mouse IgG(H+L)Secondary Antibody, HRP](https://www.thermofisher.cn/order/genome-database/details/antibody/31430?SID=srch-srp-31430) | Invitrogen™ | Cat#31430, RRID:AB_228307 |
| Donkey anti-Goat IgG(H+L)Secondary Antibody, HRP | Invitrogen™ | Cat#A15999, RRID:AB_2534673 |
| Goat anti-Rabbit IgG(H+L)Cross-Adsorbed Secondary Antibody, Alexa Fluor™488 | Invitrogen™ | Cat#A11008, RRID:AB_143165 |
| IFLUOR™ 594 CONJUGATED GOAT ANTI-RABBIT IGG GOAT POLYCLONAL ANTIBODY | HUABIO | Cat#HA1122,  RRID:AB_3714535 |
| IFLUOR™ 488 CONJUGATED GOAT ANTI-MOUSE IGG GOAT POLYCLONAL ANTIBODY (HA1125) | HUABIO | Cat#HA1125 |
